# Dr. Austin Flint, Sr.

**Published:** 1886-04

**Authors:** 


### DR. AUSTIN FLINT, SR.

With great sadness we notice the death of this most distinguished member of the medical profession in the United States. Advanced in years and covered with honors, he passed from the scene of his labors and usefulness to that of his rest and reward.

For many years Dr. Flint has enjoyed a position as eminent as it is possible for a physician in this country to attain. His reputation as a didactic teacher placed him at the head of the list; as a diagnostician, he was unsurpassed; as a clear, truthful and conscientious writer and observer of medical subjects, his was the highest authority; as a practitioner, his clear judgment and conservatism, uninfluenced by enthusiasm or partizanship, made him the safest of guides for those so fortunate as to follow his precepts.

The world is familiar with Dr. Flint's voluminous contributions to medical science. His careful research, his keen analysis, his incisive conclusions, his impartiality and truth, have shed a flood of light along the entire pathway not soon to be obscured or forgotten.

His contributions to the science of physical diagnosis are perhaps the most original and best known of his achievements, but these constitute only a part of the history of a long life-work, whose faithful performance entitled him, in the judgment of all the world, to the highest position among American physicians.

